# Decreased plasma concentrations of BDNF and IGF-1 in abstinent patients with alcohol use disorders

**DOI:** 10.1371/journal.pone.0187634

**Published:** 2017-11-06

**Authors:** Nuria García-Marchena, Daniel Silva-Peña, Ana Isabel Martín-Velasco, María Ángeles Villanúa, Pedro Araos, María Pedraz, Rosa Maza-Quiroga, Pablo Romero-Sanchiz, Gabriel Rubio, Estela Castilla-Ortega, Juan Suárez, Fernando Rodríguez de Fonseca, Antonia Serrano, Francisco Javier Pavón

**Affiliations:** 1 Unidad Gestión Clínica de Salud Mental, Instituto de Investigación Biomédica de Málaga (IBIMA), Hospital Regional Universitario de Málaga, Universidad de Málaga, Málaga, Spain; 2 Departamento de Fisiología, Facultad de Medicina, Universidad Complutense de Madrid, Madrid, Spain; 3 Instituto i+12, Hospital Universitario 12 de Octubre, Madrid, Spain; Chiba Daigaku, JAPAN

## Abstract

The identification of growth factors as potential biomarkers in alcohol addiction may help to understand underlying mechanisms associated with the pathogenesis of alcohol use disorders (AUDs). Previous studies have linked growth factors to neural plasticity in neurocognitive impairment and mental disorders. In order to further clarify the impact of chronic alcohol consumption on circulating growth factors, a cross-sectional study was performed in abstinent AUD patients (alcohol group, N = 91) and healthy control subjects (control group, N = 55) to examine plasma concentrations of brain-derived neurotrophic factor (BDNF), insulin-like growth factor-1 (IGF-1) and IGF-1 binding protein-3 (IGFBP-3). The association of these plasma peptides with relevant AUD-related variables and psychiatric comorbidity was explored. The alcohol group was diagnosed with severe AUD and showed an average of 13 years of problematic use and 10 months of abstinence at the moment of participating in the study. Regarding common medical conditions associated with AUD, we observed an elevated incidence of alcohol-induced liver and pancreas diseases (18.7%) and psychiatric comorbidity (76.9%). Thus, AUD patients displayed a high prevalence of dual diagnosis (39.3%) [mainly depression (19.9%)] and comorbid substance use disorders (40.7%). Plasma BDNF and IGF-1 concentrations were significantly lower in the alcohol group than in the control group (p<0.001). Remarkably, there was a negative association between IGF-1 concentrations and age in the control group (r = -0.52, p<0.001) that was not found in the alcohol group. Concerning AUD-related variables, AUD patients with liver and pancreas diseases showed even lower concentrations of BDNF (p<0.05). In contrast, the changes in plasma concentrations of these peptides were not associated with abstinence, problematic use, AUD severity or lifetime psychiatric comorbidity. These results suggest that further research is necessary to elucidate the role of BDNF in alcohol-induced toxicity and the biological significance of the lack of correlation between age and plasma IGF-1 levels in abstinent AUD patients.

## Introduction

Alcohol consumption is one of the most important health problems in our society [1]. Chronic alcohol consumption can affect neurons and glial cells in the central nervous system (CNS), producing profound changes in synaptic structure and function. In fact, the toxic effects of alcohol exposure during early development may result in long-lasting behavioral and neurocognitive changes [2]; and excessive alcohol exposure during adulthood may lead to a chronic relapsing brain disease that receives the medical diagnosis of alcohol use disorder (AUD) [3]. Currently, AUD is a single disorder that integrates both alcohol abuse and alcohol dependence and is characterized by compulsive alcohol use, loss of control over alcohol intake, and a negative emotional state emerging in conjunction with withdrawal and protracted abstinence [3–4]. AUD is commonly associated with cognitive and memory impairment during development with a higher incidence of comorbid mental disorders, particularly depression and anxiety [5], and other organic comorbid diseases (e.g., alcohol liver diseases) [3, 6].

Research in alcohol addiction has reported the existence of areas of the brain that may be particularly vulnerable to alcohol toxicity through interference with the activity of cell-signaling systems, particularly growth and trophic factors [7–8]. Indeed, studies in preclinical and clinical models of AUD provide evidence of the role of neurotrophic factors in alcohol-induced cognitive decline in brain areas, including the basal forebrain-cortex system [9–10].

Growth factors are a set of proteins known to play a crucial role in cellular growth, proliferation and differentiation in the CNS. These factors are produced by several cells (e.g., noradrenergic, dopaminergic, cholinergic neurons and astrocytes) and have been extensively studied because of their implications in the development of neuroadaptations in the CNS but also in the immune and endocrine systems [11–12].

Among the different families of growth factors, the neurotrophin family has received much attention for its role in neuropsychiatric conditions such as Alzheimer’s disease, Huntington’s disease and bipolar disorder [11–13]. This family is composed of neurotrophic peptides such as nerve growth factor, brain-derived neurotrophic factor (BDNF), neurotrophin 3 and neurotrophin 4. BDNF is a neurotrophic factor abundantly expressed in the CNS and in peripheral tissues acting through the trkB receptor [14]. BDNF plays a prominent role in cognitive processes such as learning, memory and behavioral consolidation [15], and alterations in BDNF concentration can be observed in mental disorders, particularly with depression [16]. In fact, the presence of depressive disorders is associated with lower levels of trophic support in clinical and preclinical models [16–18]. Regarding drug addiction, BDNF reduces the motivation and behavioral effects of alcohol consumption and is associated with decreased alcohol consumption [19]. In alcohol-dependent patients, serum BDNF has been reported to be associated with withdrawal severity (i.e., somatic symptoms, tolerance, craving and alcohol consumption to avoid withdrawal symptoms) [20].

Insulin-like growth factors (IGFs) are another family of proteins that are part of a complex signaling system that includes IGFs (i.e., IGF-1 and IGF-2), receptors, IGF-binding proteins and proteases. IGF-1 is cytoprotectant [21] and changes in this peptide signaling may be involved in the pathogenesis of neurological and psychiatric disorders, including anxiety and depression [22–23]. In clinical models, IGF-1 has been related to the persistence of associated memories related to drug reinforcement in alcohol craving and opiate dependence [24–25]. Associated with IGF-1, IGFBP-3 is a binding protein in humans that prolongs the half-life of IGF-1 and, with respect to the deleterious effects of alcohol, IGFBP-3 may be involved in the length of alcohol abstinence [26].

One interesting aspect is the potential evaluation of these factors in the plasma because they circulate in the blood and can cross the blood-brain barrier to exert their effects [27]. Therefore, although much work is still needed to identify the effect of chronic alcohol consumption on those growth factors regulating the functional integrity of the CNS, it is feasible to hypothesize that AUDs are associated with alterations in the circulating levels of main growth factors.

Because the search for peripheral biomarkers of AUD has increased notably over the last few years, we designed an exploratory and descriptive cross-sectional study in a cohort of abstinent alcohol-addicted patients from outpatient treatment programs to examine plasma concentrations of BDNF, IGF-1 and IGFBP-3. Consequently, the main objective of this study was to explore associations between these plasma peptides and relevant AUD-related variables (i.e., alcohol abstinence, problematic alcohol use, alcohol symptom severity, alcohol-induced liver and pancreas diseases) and psychiatric comorbidity, which were established using psychiatric and clinical assessments.

## Materials and methods

### Subjects and recruitment

The present cross-sectional study included 146 participants (White Caucasian) divided into two groups: i) an alcohol group (91 patients with AUD) and ii) a control group (55 healthy control subjects).

The initial procedure for recruiting was non-random convenience sampling according to the participation criteria, which were established at the beginning of the study: Ninety-five patients were initially recruited from active outpatient treatment programs in *Hospital Universitario 12 de Octubre* (Madrid, Spain) for a period of 18 months (from November 2013 to May 2015); sixty control individuals were recruited from multidisciplinary staff working at the *Hospital Universitario 12 de Octubre* and *Universidad Complutense de Madrid* (Madrid, Spain) and matched for age, sex and body mass. The eligibility of all participants was subsequently corroborated using both clinical assessments and plasma tests, and 9 participants were excluded from the study.

#### Participation criteria

The participation was absolutely voluntary, but all participants had to meet eligibility criteria based on inclusion and exclusion criteria for the study.

*Alcohol group*. Inclusion criteria included the following: age ≥ 18 years up to 65 years of age, lifetime AUD diagnosis and at least 4 weeks of abstinence. The exclusion criteria of the alcohol group were as follows: the presence of infectious diseases, personal history of cancer, incapacitating cognitive alterations and pregnancy. Four abstinent AUD patients were excluded from the initial cohort according to the exclusion criteria.

*Control group*. Control subjects were matched for sex, age and body mass with abstinent AUD patients and they were required to be ≥18 years to 65 years of age. The exclusion criteria were personal history of drug abuse and/or dependence (i.e., lifetime AUD or other substance use disorders), lifetime psychiatric disorders, the presence of infectious diseases, personal history of cancer, incapacitating cognitive alterations and pregnancy. Five control subjects were excluded from the study.

### Ethics statement

Written informed consent was obtained from each participant after a complete description of the study. All participants had the opportunity to discuss any questions or issues. The study and protocols for recruitment were approved by the Ethics Committee of the *Hospital Regional Universitario de Málaga* (CP14/00173 and PI13/02261) in accordance with the “Ethical Principles for Medical Research Involving Human Subjects” adopted in the Declaration of Helsinki by the World Medical Association (64th WMA General Assembly, Fortaleza, Brazil, October 2013), Recommendation No. R (97) 5 of the Committee of Ministers to Member States on the Protection of Medical Data (1997), and the Spanish Data Protection Act (*Ley Orgánica 15/1999 de Protección de Datos*, LOPD). All collected data were coded to maintain privacy and confidentiality.

### Clinical assessments

Prior to the clinical assessments, the “Trail Making Test” (TMT) Part B was easily administered to all participants as a memory and attention-screening test to detect cognitive alterations [28]. The primary psychiatric assessment tool was the Spanish version of the “Psychiatric Research Interview for Substance and Mental Diseases” (PRISM) according to “Diagnostic and Statistical Manual of mental Disorders-4^th^ Edition-Text Revision” (DSM-IV-TR) criteria [29–30]. This semi-structured interview has demonstrated good psychometric properties in terms of test-retest reliability and inter-rater reliability and validity. Lifetime prevalence was used to present the frequency of substance use disorders and other mental disorders, taking into account “current” (criteria were met within the past year) and “past diagnoses” (criteria were met before the previous 12 months). The control group was evaluated by the Spanish version of the “Composite International Diagnostic Interview” (CIDI) to detect psychiatric disorders [31].

Alcohol symptom severity was assessed by combining the DSM-IV-TR criteria for alcohol abuse and dependence: 4 abuse criteria (one symptom is necessary for diagnosis of abuse); and 7 dependence criteria (for diagnosis of dependence three or more co-occurring symptoms in a 12-month period are required) [32].

Psychologists who had received PRISM and CIDI training performed all the interviews.

### Collection of plasma samples and rapid analyses

Blood samples were obtained in the morning (9:00–11:00 a.m.) after fasting for 8–12 h (previous to the psychiatric interviews). Venous blood was extracted into 10mL K_2_ EDTA tubes (BD, Franklin Lakes, NJ, USA) and was immediately processed to obtain plasma. All blood samples were centrifuged at 2,200 x g for 15 min (4°C) and individually assayed for detecting infectious diseases by three commercial rapid tests for HIV, hepatitis B, and hepatitis C [33]. Samples displaying infection were discarded following safety protocols. Additionally, the blood alcohol concentration was measured according to the alcohol oxidase reaction using an Analox AM1 analyzer (Analox Instruments, Stourbridge, UK).

Finally, plasma samples were individually characterized, registered (code number), and stored at -80°C until further analyses.

### Multiplex immunoassays for BDNF

A Bio-Plex Suspension Array System 200 (Bio-Rad Laboratories, Hercules, CA, USA) was used to quantify the plasma concentrations of growth factors and other related peptides [34]. Human protein panels were used to detect the BDNF levels and other analytes that were recently published [33]. However, commercially available human ELISA kits are unable to distinguish between precursors of BDNF and mature BDNF because of limited BDNF antibody specificity [35]. Raw data (mean fluorescence intensity) were analyzed using the Bio-Plex Manager Software 4.1 (Bio-Rad laboratories, Hercules, CA, USA). BDNF concentrations were expressed as pg/mL.

### Radioimmunoassay analysis for IGF-1 and IGFBP-3

As previously described [32], plasma concentrations of total IGF-1 were estimated by double antibody radioimmunoassay (RIA), after removal of serum IGFBPs by acid-ethanol extraction. To confirm the removal of IGFBPs, extracted and non-extracted plasma fractions were incubated with ^125^I-IGF-1 at 4°C for 24 h. Dextran charcoal was used to separate the bound and free tracers. The IGF-1 antiserum (UB2-495) was a gift from Drs. Underwood and Van Wisk distributed by the Hormone Distribution Program of the National Institute of Diabetes and Digestive and Kidney Diseases (NIDDK) through the National Hormone and Pituitary Program. Concentrations of IGF-1 were expressed in terms of rat IGF-1 from Gropep Bioreagents Pty Ltd. (Adelaide, SA, Australia) as ng/mL. A plasma IGFBP-3 concentration was determined in duplicate by RIA using a commercially available kit (Mediagnost GmbH, Reutlinger, Germany) following the manufacturer’s instructions. IGFBP-3 concentrations were expressed as μg/mL.

### Statistical analysis

All data in tables are expressed as the number and percentage of subjects [N (%)] or mean and standard deviation (SD) of concentrations [mean (SD)]. The significance of differences in categorical and normal continuous variables was determined using Fisher’s exact test (chi-square test) and Student’s *t*-test, respectively.

Statistical analysis of BDNF, IGF-1 and IGFBP-3 concentrations was performed using analysis of covariance (ANCOVA) as a general linear model to indicate the relative effect of explanatory variables and their interactions on the expression of these peptides in plasma, controlling for covariates. Therefore, the ANCOVA models included AUD-related variables as categorical and continuous independent variables to explore their association with plasma BDNF, IGF-1 and IGFBP-3 concentrations. Prior to the selection and inclusion of independent variables in these analyses, the ANCOVA assumptions regarding the independence of covariates and homogeneity of regression slopes were evaluated based on parametric assumptions. Log (10)-transformation for peptide concentrations was used to ensure parametric assumptions for positively skewed distributions and estimated marginal means [95% confidence intervals (95% CI)] were expressed and represented in the figures after back-transformation when those criteria were met. *Post hoc* comparisons were performed using Sidak’s correction test.

In addition, correlation analyses between peptide concentrations and independent variables were performed using the Pearson’s correlation coefficient (r).

A p-value <0.05 was considered statistically significant. Statistical analyses were performed using the GraphPad Prism version 5.04 (GraphPad Software, San Diego, CA, USA) and IBM SPSS Statistical version 22 software (IBM, Armonk, NY, USA).

## Results

### Sample demographics

This study included 146 participants, who were divided into alcohol (N = 91) and control (N = 55) groups. **Table 1** shows a socio-demographic description of the sample. The average participant in the alcohol group was a 48-year-old male with a BMI of 26. Significant differences were observed between the two groups with respect to marital status (p<0.01), education (p<0.05), occupation (p<0.001) and medication use (p<0.001). Thus, the alcohol group was characterized by a higher prevalence of marital separation, a lower education level and a higher unemployment rate relative to the control group. Unlike the control participants, abstinent AUD patients had taken psychotropic medication in the last year: 44% antidepressants, 32% anxiolytics, 33% anticonvulsants and 9% antipsychotics. In addition to psychiatric medication use, 67 AUD patients were treated with *disulfiram* in the last 12 months.

**Table 1:** Baseline socio-demographic variables.

Variable		Alcohol group (N = 91)	Control group (N = 55)	p-value
**Age** *[mean (SD)]*	*Years*	47.91 (7.69)	45.15 (10.06)	0.083 ^a^
**BMI** *[mean (SD)]*	*kg/m*^*2*^	25.86 (3.89)	24.82 (3.37)	0.090 ^a^
**Sex** *[N (%)]*	Women	30 (33.0)	19 (34.5)	0.847 ^b^
	Men	61 (67.0)	36 (65.5)	
**Marital status** *[N (%)]*	Single	29 (31.8)	29 (52.7)	<0.01 ^b^
	Cohabiting	32 (35.2)	21 (38.2)	
	Separated	28 (30.8)	5 (9.1)	
	Widow	2 (2.2)	-	
**Education** *[N (%)]*	Elementary	31 (34)	6 (10.9)	<0.05 ^b^
	Secondary	32 (35.2)	16 (29.1)	
	University	28 (30.8)	33 (60.0)	
**Occupation** *[N (%)]*	Employed	48 (52.7)	51 (92.7)	<0.001 ^b^
	Unemployed	43 (47.3)	4 (7.3)	
**Psychiatric medication use** *[N (%)]*	No	26 (28.6)	64 (100)	<0.001 ^b^
	Yes	65 (71.4)	-	

Abbreviations: BMI = body mass index

^a^ p-value from Student’s t-test

^b^ p-value from Fisher’s exact test or chi-square test.

### Plasma concentrations of BDNF, IGF-1 and IGFBP-3 in relation to alcohol use disorders

**Fig 1:**
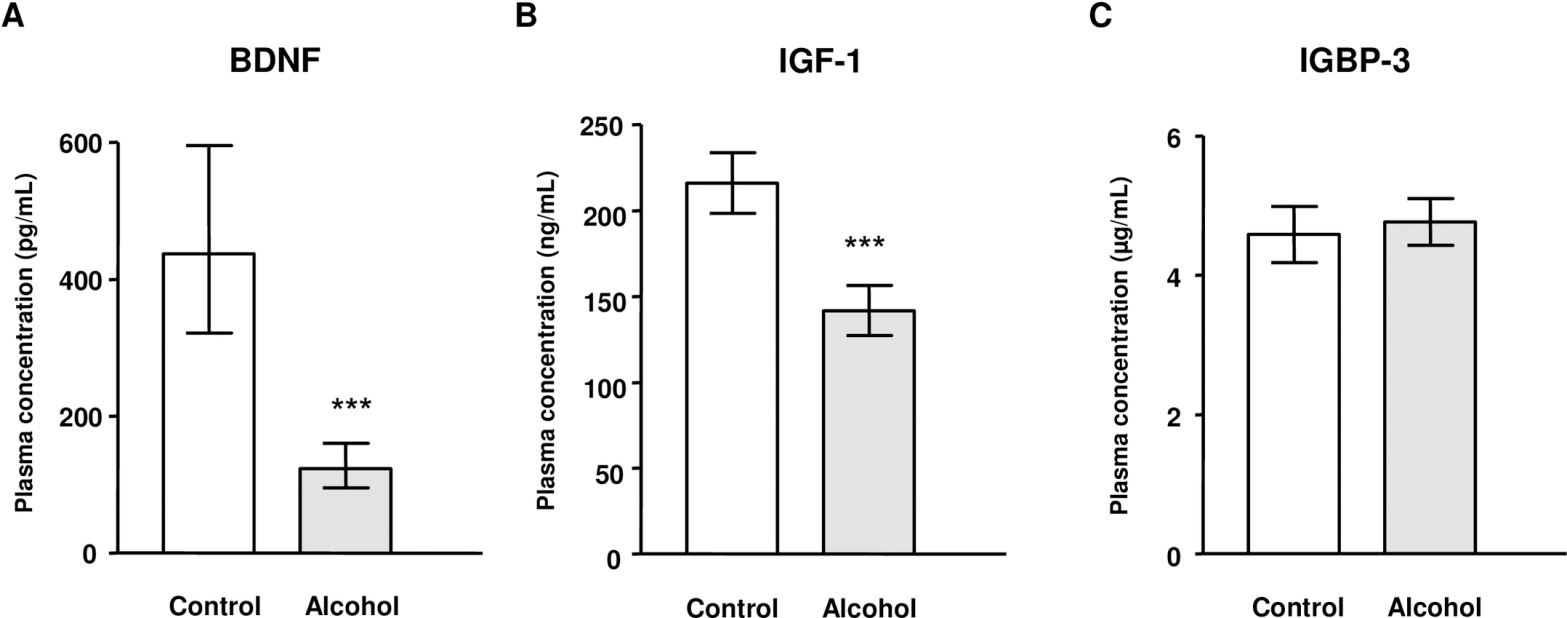
Plasma concentrations of BDNF, IGF-1 and IGFBP-3 in the alcohol and control groups. (A) BDNF (pg/mL); (B) IGF-1 (ng/mL); and (C) IGFBP-3 (μg/mL). Bars are the back-transformed means and 95% CI from logarithmic data. (***) p<0.001 denotes significant differences between the alcohol and control groups.

The effect of AUD on the plasma concentrations of BDNF, IGF-1 and IGFBP-3 in male and female participants (N = 97 men and N = 49 women) was investigated using a two-way ANCOVA (“lifetime AUD” and “sex” as factors), controlling for “age” and “BMI”. Data were examined, and a base-10 logarithmic transformation of the concentrations of BDNF, IGF-1 and IGFBP-3 was performed. Back-transformed marginal means on the original scale are shown in **Fig 1**. We observed different effects of the independent variables on the expression of both growth factors and IGFBP-3 in plasma.

The statistical analysis revealed a main effect of “lifetime AUD” on BDNF concentrations (F_(1,138)_ = 39.597; p<0.001) (**Fig 1A**). This effect resulted in decreased levels of BDNF in the alcohol group compared with those measured for the control group [127.35 (95% CI = 98.17–165.19) pg/mL and 483.05 (95% CI = 348.33–671.42) pg/mL, respectively], with no effect of “sex” or interaction.

Similarly, the plasma concentrations of IGF-1 were significantly affected by “lifetime AUD” (F_(1,138)_ = 42.522; p<0.001) (**Fig 1B**). Thus, the alcohol group had lower levels of IGF-1 than the control group [140.42 (95% CI = 125.89–154.95) ng/mL and 218.62 (95% CI = 199.99–237.26) ng/mL, respectively]. There was no effect of “sex” or interaction between factors on IGF-1 concentrations. In addition to the “lifetime AUD” effect, plasma IGF-1 concentrations were strongly affected by the covariate “age” (F_(1,138)_ = 14.303; p<0.001).

Finally, the plasma concentrations of IGFBP-3 were not affected by any of the factors examined, “lifetime AUD” or “sex”, and there was no interaction effect (**Fig 1C**).

### Correlation analysis between age and plasma concentrations of BDNF, IGF-1 and IGFBP-3

**Fig 2:**
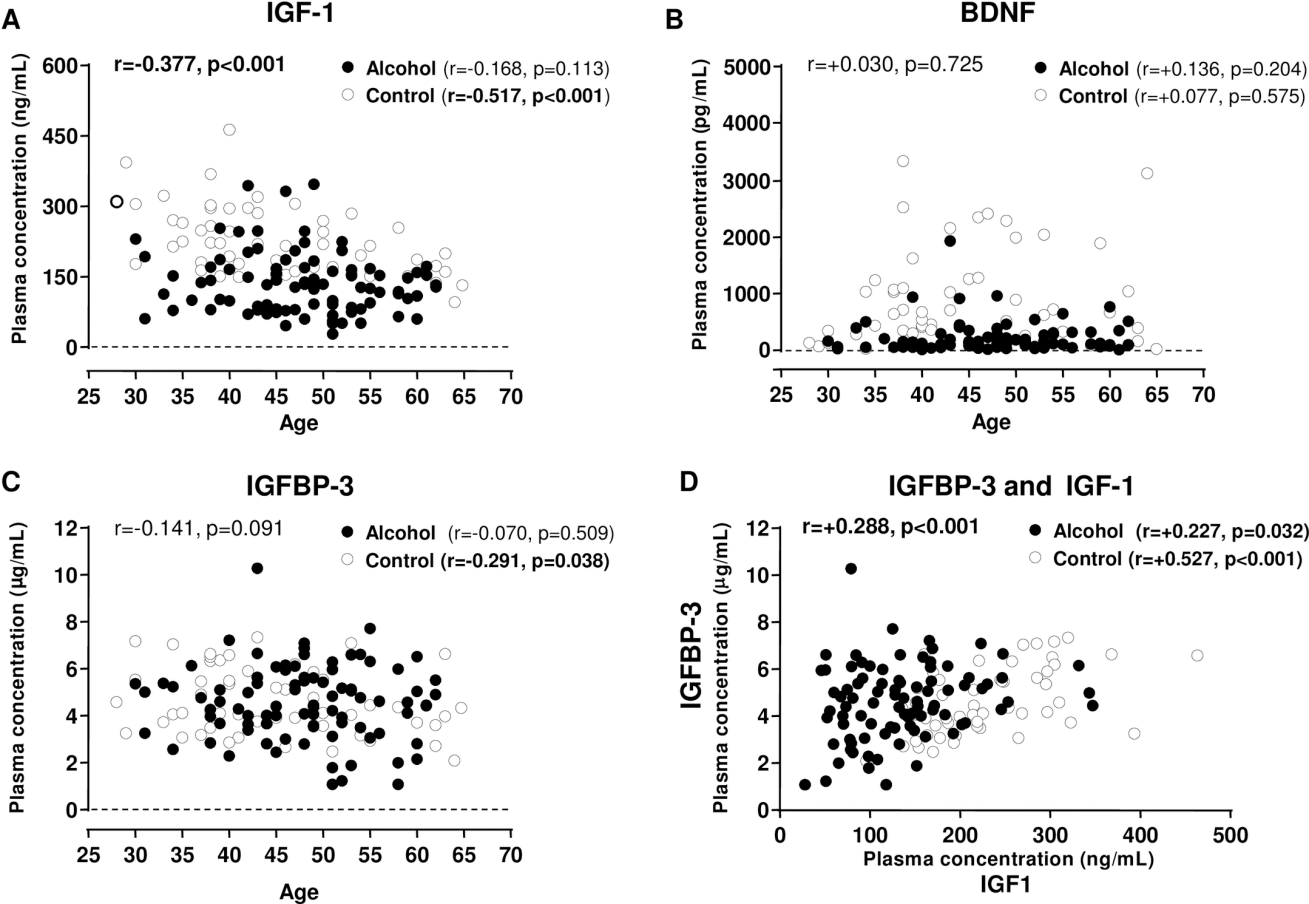
Correlation analysis between age and plasma concentrations of IGF-1, BDNF and IGFBP-3. (A) IGF-1 (ng/mL) and age (years) in the total sample; (B) BDNF (pg/mL) and age (years) in the total sample; (C) IGFBP-3 (μg/mL) and age (years) in the total sample; (D) IGF-1 (ng/mL) and IGFBP-3 (μg/mL) in the total sample. Dots represent individual raw values. The Pearson correlation coefficient (r) and p-value are indicated for statistical significance. Note: One outlier value (4.313 pg/mL and 60 years old) was not represented.

Because IGF-1 concentrations were significantly affected by age, we examined the relationship between age and plasma concentrations of this peptide in the total sample using correlation analysis (**Fig 2**).

As shown in **Fig 2A**, there was a negative correlation between age and IGF-1 concentrations (r = -0.377, p<0.001) in the total sample. When both groups were separately examined, we found a strong association between age and IGF-1 in the control group (r = -0.517, p<0.001) but not in the alcohol group. Additional correlation studies revealed that age was not associated with the plasma concentrations of BDNF (**Fig 2B**) and IGFBP-3 (**Fig 2C**). However, we found a significant correlation between age and IGFBP-3 concentrations (r = -0.291, p<0.05) in the control group.

Finally, we analyzed the relationship between the concentrations of IGF-1 and IGFBP-3. There was a positive correlation (r = +0.288, p<0.001) in the total sample (**Fig 2D**) that was also observed in both groups: r = +0.527, p<0.001 in the control group; and r = +0.227, p<0.05 in the alcohol group.

### Clinical characteristics of abstinent patients with alcohol use disorders

Abstinent AUD patients participating in the present study were clinically characterized with relevant variables associated with the diagnosis of lifetime AUD (**Table 2**). Thus, the alcohol group showed an average of 10.5 months of alcohol abstinence and 13.4 years of problematic alcohol use at the moment of evaluation. Regarding alcohol symptom severity, the alcohol participants were diagnosed with an average of 6.9 DSM-IV-TR criteria for alcohol abuse and dependence, which corresponds to severe AUD.

**Table 2:** Variables associated with AUD and psychiatric comorbidity in the alcohol group.

Variable		Alcohol group (N = 91)
**Alcohol abstinence** *[mean (SD)]*	*months*	10.5 (15.3)
**Problematic alcohol use** *[mean (SD)]*	*years*	13.4 (7.4)
**Severity of AUD** *[mean (SD)]*	*DSM-IV-TR criteria*	6.86 (2.27)
**Liver and pancreas diseases** *[N (%)]*	No	74 (81.3)
	Yes	17 (18.7)
**Other lifetime substance use disorders** *[N (%)]*	No	54 (59.3)
	Yes	37 (40.7)
	Cocaine	27 (29.7)
	Cannabis	15 (16.5)
	Sedatives	3 (3.3)
	Stimulants	6 (6.6)
**Other lifetime mental disorders** *[N (%)]*	No	24 (26.4)
	Yes	67 (73.6)
	Mood	47 (51.6)
	Anxiety	17 (18.7)
	Psychotic	6 (6.6)
	Borderline Personality	15 (16.5)
	Antisocial Personality	7 (7.7)

Because liver and pancreas diseases are common and identifiable alcohol-associated health problems among subjects with AUD, we did not exclude these patients in the present study. Specifically, 8 abstinent AUD patients were previously diagnosed with steatosis, 5 patients with cirrhosis and 4 patients with pancreatitis. These AUD patients with alcohol-related diseases (6 women and 11 men) showed an average of 5.3 (SD = 3.2) months of alcohol abstinence, 15.6 (SD = 8.1) years of problematic alcohol use and 6.7 (SD = 2.3) DSM-IV-TR criteria for alcohol abuse and dependence.

#### Psychiatric comorbidity

In addition to these variables, we found a high prevalence of lifetime psychiatric comorbidity in the alcohol group (76.9%). We distinguished between comorbid substance use disorders and mental disorders or dual diagnosis for better characterization.

Regarding comorbid substance use disorders, the highest prevalence was observed for cocaine (73%) and cannabis (41%). AUD patients with comorbid substance use disorders (7 women and 30 men) showed an average of 9.7 (SD = 11.8) months of alcohol abstinence, 13.1 (SD = 7.1) years of problematic alcohol use and 7.5 (SD = 2.8) DSM-IV-TR criteria for alcohol abuse and dependence. Five comorbid AUD patients (13.5%) had alcohol-related liver and pancreas diseases. Interestingly, these comorbid AUD patients showed an increased prevalence of dual diagnosis (81%), mainly mood disorders (57%) and personality disorders (41%).

According to comorbid mental disorders, the highest prevalence was observed for mood disorders (77%). AUD patients with dual diagnosis (23 women and 38 men) showed an average of 13.4 (SD = 17.9) months of alcohol abstinence, 13.0 (SD = 6.7) years of problematic alcohol use and 7.0 (SD = 2.4) DSM-IV-TR criteria for AUD. In this case, 8 comorbid AUD patients (13.1%) had alcohol-related liver and pancreas diseases.

### Plasma concentrations of BDNF, IGF-1 and IGFBP-3 in relation to variables related to alcohol use disorders

**Fig 3:**
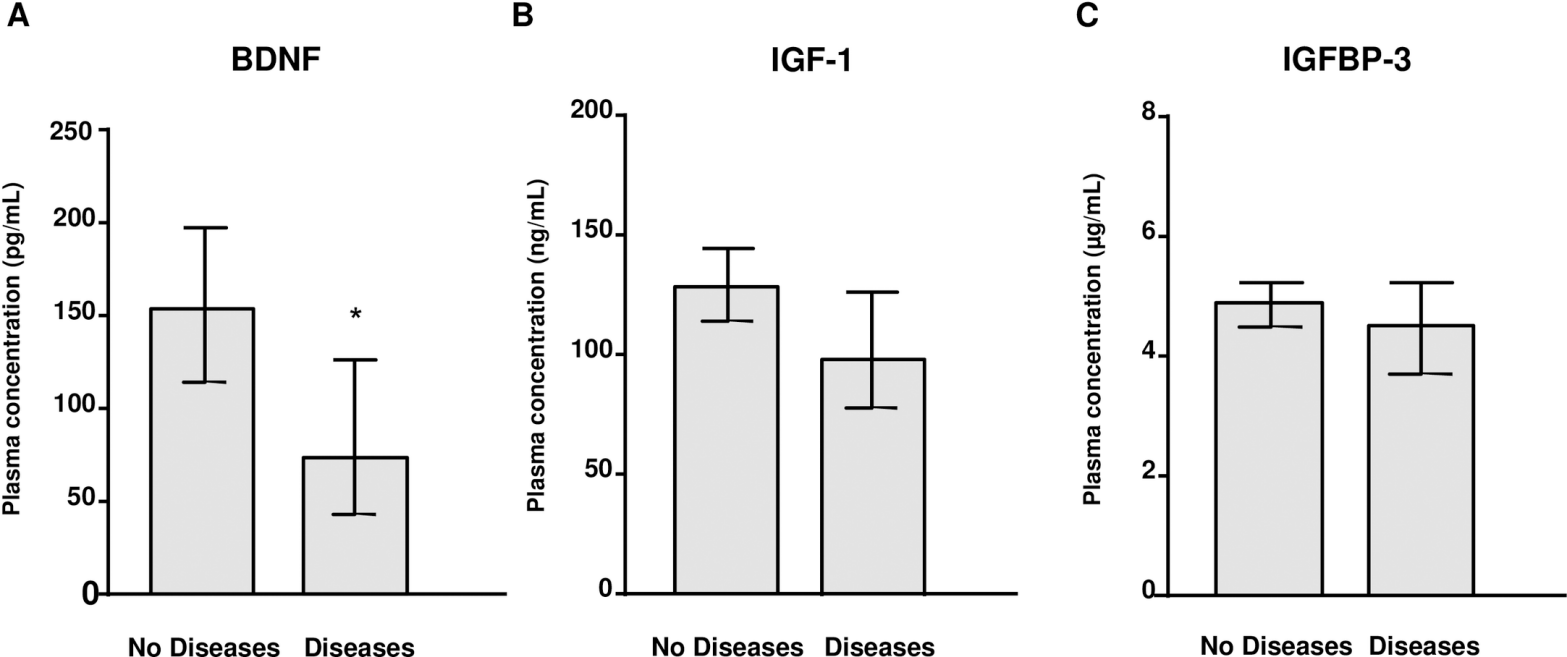
Plasma concentrations of BDNF, IGF-1 and IGFBP-3 in the alcohol group according to alcohol-induced liver and pancreas diseases. (A) BDNF (pg/mL); (B) IGF-1 (ng/mL); and (C) IGFBP-3 (μg/mL) in alcohol group. Bars are the back-transformed means and 95% CI from logarithmic data. (*) p<0.05 denotes significant differences between the disease and no-disease subgroups.

We evaluated the effects of variables associated with AUD on the plasma concentrations of BDNF, IGF-1 and IGFBP-3 in the alcohol group using an ANCOVA, controlling for age. We selected the duration of alcohol abstinence (“abstinence”), the duration of problematic alcohol use (“problematic use”) and the prevalence of alcohol-induced liver and pancreas diseases (“alcohol-induced diseases”) as variables of interest in our analyses. Back-transformed marginal means on the original scale are shown in **Fig 3**.

The statistical analysis revealed a main effect of “alcohol-induced diseases” on BDNF concentrations (F_(1,84)_ = 5.751, p = 0.019) (**Fig 3A**). In fact, abstinent AUD patients with liver and/or pancreas diseases had lower BDNF concentrations relative to AUD patients with no disease [74.41 (95% CI = 43.77–126.51) pg/mL and 152.18 (95% CI = 118.15–196.03), respectively]. In contrast, the plasma concentrations of IGF-1 and IGFBP-3 were not significantly affected by “alcohol-induced diseases” (**Fig 3B** and **Fig 3C**, respectively).

Regarding “abstinence” and “problematic use”, we observed no significant effects on plasma concentrations of BDNF, IGF-1 or IGFBP-3. Similarly to previous analyses in the total sample, plasma concentrations of IGF-1 were significantly affected by the covariate “age” (F_(1,85)_ = 5.253; p = 0.024).

#### Plasma concentrations of BDNF, IGF-1 and IGFBP-3 in relation to psychiatric comorbidity

Because of the high incidence of psychiatric comorbidity, we analyzed the effects of this additional psychiatric condition on the plasma concentrations of BDNF, IGF-1 and IGFBP-3 using ANCOVA. The model also included “alcohol-induced diseases”, controlling for “medication use” and “age”. Complementarily, we distinguished between comorbid substance use disorders and comorbid mental disorders.

However, we found no significant effects of psychiatric comorbidity on the expression of these peptides in the plasma of abstinent AUD patients. Back-transformed marginal means are shown in **S1 Table**.

## Discussion

In the present study, we examined BDNF, IGF-1 and IGFBP-3 concentrations in the plasma of abstinent AUD patients, who were recruited from active outpatient treatment programs, and healthy subjects. Additionally, these abstinent patients were clinically characterized through psychiatric interviews based on the DSM-IV-TR, and main AUD-related variables were determined (i.e., alcohol abstinence, problematic alcohol use, alcohol symptom severity, alcohol-induced liver and/or pancreas diseases and psychiatric comorbidity). The association of these plasma peptides and AUD-related variables was analyzed using ANCOVA models to identify potential biomarkers of lifetime AUD.

The main findings are as follows: a) Plasma concentrations of BDNF and IGF-1 were significantly lower in the alcohol group than in the control group; b) there was a strong correlation between IGF-1 concentrations and age in the control group that was not observed in the alcohol group; c) AUD patients with alcohol-induced liver and/or pancreas diseases showed even lower concentrations of BDNF than other AUD patients; d) in contrast, BDNF, IGF-1 and IGFBP-3 concentrations were not statistically affected by other AUD-related variables such as abstinence, problematic alcohol use, alcohol symptom severity or psychiatric comorbidity.

### Plasma concentrations of BDNF and IGF-1are altered in abstinent patients with alcohol use disorders

Because of their role in CNS development, growth factors have been studied in neuroplasticity modulation and behavioral effects derived from alcohol-induced toxicity [12, 36–37]. For instance, nerve growth factor (NGF) is a growth factor linked to inflammatory and endocrine responses that has been reported to be altered in the blood of alcohol-dependent patients [38–39]. Our data pertaining to BDNF and IGF-1 support growth factors could be linked to alcohol dependence. Thus, the decreased levels of BDNF and IGF-1 in patients with AUD that we observed are in accord with other studies in alcohol, which have reported an association between decreased BDNF levels and alcohol intoxication [36] and between inhibited IGF-1 bioavailability and chronic alcohol abuse [40]. However, we found no associations between these decreased levels in plasma and relevant variables associated with AUD, such as abstinence, problematic alcohol use or severity of the AUD.

The long-term persistence of the alcohol-induced inhibition of circulating BDNF and IGF-1 is remarkable because our AUD cohort had an average of 10 months of abstinence. In fact, we found no association between these peptides and the duration of abstinence. In contrast, other studies have shown that BDNF and IGF-1 levels are altered differently by the duration of abstinence. Thus, a decrease in plasma BDNF concentrations has been reported after one month of hospitalization [41], in line with the trend indicated by our data. In contrast, other studies in alcohol populations with shorter durations of abstinence describe elevated concentrations of BDNF within the first 24 h after admission [42] and one week after alcohol withdrawal [43]. Interestingly, while D'sa and colleagues observed higher BDNF levels in the serum of alcohol-dependent subjects with 4 weeks of controlled abstinence relative to social drinkers, no changes in plasma BDNF levels were reported [44]. Regarding IGF-1, a clinical study with alcohol-addicted patients reported low levels of serum IGF-1 relative to those measured in non-addicted subjects, but these levels increased after 2–5 days of abstinence [40]. Because we have not studied the course of acute abstinence but rather plasma concentrations in a cross-sectional design, our data cannot be compared with those reflecting transient increments during abstinence. Furthermore, there are additional differences in experimental design among studies concerning the blood compartment that is analyzed (e.g., plasma *vs*. serum) and the treatment programs examined (e.g., outpatient *vs*. inpatient).

### The association between plasma IGF-1 levels and age is not observed in abstinent patients with alcohol use disorders

Growth factors are important in the maintenance of the neural homeostatic conditions in the CNS during aging [45]. Thus, the neuroprotective activity of growth factors has been extensively studied, and reports have revealed that the expression of peptides such as IGF-1 is related to life span and longevity [46]. In fact, IGF-1 function is decreased in the brain during aging [47]. Accumulating evidence reveals that circulating levels of IGF-I also significantly affect cognitive brain function and are reduced in the elderly [48]. In agreement, we previously reported that plasma IGF-1 concentrations are negatively correlated with age in an exploratory study with cocaine users and healthy subjects [32], with no effects of chronic cocaine consumption on the expression of this growth factor. In the present study, we also found a strong correlation between plasma IGF-1 and age in the control group, but unlike in the cocaine study, there was no association between both variables in abstinent AUD patients. Our data reveal that subjects with chronic alcohol consumption (13 years of problematic use) show lower IGF-1 concentrations than those of control subjects within the same age range. These findings are concordant with the fact that chronic excessive alcohol consumption induces cognitive impairments [49].

### Plasma BDNF levels are decreased in abstinent patients with alcohol-induced liver and/or pancreas diseases

Growth factor deficiencies have been linked to oxidative and inflammatory states in vascular dysfunctions, diabetes and other important diseases in accessory organs [50–51]. In fact, active drinking with AUD and the presence of an intense inflammatory response are associated with organ failure and high short-term mortality [52].According to our results, the prevalence of AUD participants with alcohol-induced diseases in accessory organs (i.e., liver and pancreas) was 19%, and these patients showed lower BDNF concentrations. We found no study in the literature about the association between BDNF expression in the blood and alcoholic pancreatitis or alcoholic liver disease, and only some studies have associated alterations in BDNF with non-alcoholic chronic pancreatitis [53] and non-alcoholic hepatitis [54]. Furthermore, BDNF concentrations has been reported to be associated with impairment of physical aspects of quality of life in patients with hepatitis C, and this association is independent of drug abuse status, mental problems, treatment or cirrhosis severity [54]. Although IGF-1 and IGBP-3 have been reported to be altered in previous studies performed in atherosclerosis and other cardiovascular diseases, we found no significant differences between the two peptides among patients with hepatitis, cirrhosis or pancreatitis [55–56].

### Plasma BDNF, IGF-1 and IGFBP-3 levels are not associated with psychiatric comorbidity

Numerous studies have suggested that growth factors play important roles in psychiatric disorders. Thus, BDNF has been found to be reduced in depression and anxiety disorders in clinical studies [57–58], while IGF-1 has been associated with the etiology of depression [59], particularly in learning and memory deficits, correlating with fatigue, tension and anxiety [60–61]. In our alcohol group, we found a high prevalence of psychiatric comorbidity [dual diagnosis (39%), e.g., depression (20%), anxiety (7%) and borderline personality disorders (7%); and comorbid substance use disorders (41%), e.g., cocaine (30%) and cannabis (17%)], but we observed no association between plasma concentrations of these peptides and the diagnosis of psychiatric comorbidity. In contrast, dysregulation of growth factors in the CNS has been associated with comorbid mental disorders, particularly BDNF and NGF [37, 62]. In fact, we reported an association between BDNF concentrations and comorbid mood disorders in patients with cocaine use disorders [32].

### Limitations and future perspectives

This exploratory study examined the effects of AUD-related variables on the expression of main growth factors in the plasma of abstinent AUD patients to identify and explore new molecular tools and potential biomarkers in the pathogenesis of drinking problems, in particular, AUD and alcohol-induced medical complications.

Although our findings support the importance of monitoring growth factors in the context of AUD and related diseases, we are aware of the limitations of this cross-sectional study. First, we must consider that unlike inpatient treatment programs, the recruitment and selection of the sample was conducted from outpatient programs. Second, the validity of the present results must be assessed in a larger sample of AUD patients, and additional experimental groups should be included (e.g., subjects with mental disorders but with no substance use disorders, active alcohol-drinking subjects). Third, a longitudinal study is also needed to monitor changes in growth factors according to the duration of alcohol abstinence in the same patients. Complementarily, preclinical studies will be able to elucidate the potential association between these circulating peptides and abstinence. Fourth, there is a growing number of socio-demographic variables that could influence the expression of growth factors, but they were not included in the present analyses because of the relative small sample size, statistical restrictions and differences in the source of recruitment.

In conclusion, these findings support that alterations in circulating growth factors are associated with a personal history of AUD. Monitoring circulating growth factors such as BDNF and IGF-1 could help stratify subjects with AUD during the different phases of alcohol detoxification programs and predict treatment outcomes. Further investigation is needed to explore the potential role of BDNF as an indicator or biomarker of inflammatory pancreas and liver diseases associated with chronic alcohol consumption. Finally, the biological significance of the lack of correlation between age and plasma IGF-1 levels in abstinent AUD patients must be investigated.

## Supporting information

S1 Table Plasma concentrations of BDNF, IGF-1 and IGFBP-3 in the alcohol group according to psychiatric comorbidity. (PDF)

S1 File Database. (XLSX)
