# Supplementary material for: Decreased plasma concentrations of BDNF and IGF-1 in abstinent patients with alcohol use disorders
Source: PLoS One. 2017 Nov 6;12(11):e0187634. doi: 10.1371/journal.pone.0187634 (PMC5673472; doi:10.1371/journal.pone.0187634)
Supplement: S1 Table — (PDF) [file pone.0187634.s001.pdf]

S1 Table. Plasma concentrations of BDNF, IGF-1 and IGFBP-3 in the alcohol group according to psychiatric comorbidity

Variable	Alcohol (N=91)					
	Psychiatric comorbidity		Comorbid substance use disorders		Comorbid mental disorders	
	**No** (N=20)	**Yes** (N=71)	**No** (N=54)	**Yes** (N=37)	**No** (N=30)	**Yes** (N=61)
**BDNF (*)** *[mean (95% CI)]*	121.14 (72.70-201.86)	108.13 (78.00-149.89)	98.00 (68.71-137.21)	139.62 (91.25-213.63)	125.41 (78.86-199.44)	105.02 (74.63-147.78)
**IGF-1** *[mean (95% CI)]*	103.61 (82.55-130.05)	113.00 (97.40-131.09)	103.05 (88.06-120.58)	123.38 (102.21-148.93)	109.62 (89.08-134.89)	110.94 (94.86-129.75)
**IGFBP-3** *[mean (95% CI)]*	4.22 (3.48-4.97)	4.65 (4.16-5.14)	4.40 (3.87-4.92)	4.77 (4.13-5.39)	4.41 (3.73-5.10)	4.60 (4.09-5.12)

ANCOVA revealed no significant main effects or interaction using comorbidity and sex as factors and controlling for age
Data represented in the table are the estimated marginal means and 95%CI for levels of comorbidity.
(*) BDNF concentrations are the back-transformed means and 95% CI from logarithmic data.
